# Impact of Cold Stress on Hepatopancreas Transcriptomic and Metabolomic in Red Swamp Crayfish *Procambarus clarkii*

**DOI:** 10.3390/ijms26031221

**Published:** 2025-01-30

**Authors:** Xiaochen Zhu, Aidi Peng, Yueying Zou, Yingdong Li, Hua Wei, Xianhu Zheng, Yingying Zhao

**Affiliations:** 1 Hebei Key Laboratory of the Bohai Sea Fish Germplasm Resources Conservation and Utilization, Beidaihe Central Experiment Station, Chinese Academy of Fishery Sciences, Qinhuangdao 066100, China; 2 College of Animal Science and Veterinary Medicine, Shenyang Agricultural University, Shenyang 110866, China; 3 College of Science and Engineering, Flinders University, Adelaide, SA 5042, Australia; 4 Key Laboratory of Freshwater Aquatic Biotechnology and Breeding, Ministry of Agriculture and Rural Affairs, Heilongjiang Fisheries Research Institute, Chinese Academy of Fishery Sciences, Harbin 150070, China

**Keywords:** *Procambarus clarkii*, cold stress, hepatopancreas, transcriptomic, metabolomic

## Abstract

The aquaculture industry of red swamp crayfish (RSC), *Procambarus clarkii*, has grown significantly in recent decades due to increasing market demand. In China, low water temperatures, particularly during overwintering, pose a challenge, hindering the development of the RSC aquaculture industry in northern regions. Understanding the molecular mechanism of RSCs’ responses to cold stress could be beneficial for its aquaculture practices. In this study, we exposed RSCs to 4 °C (T4) and 22 °C (T22: control) for 96 h. Transcriptomic and metabolomic analyses of hepatopancreas tissues were performed to identify key genes and metabolites that participate in cold stress response. A total of 787 differentially expressed genes (DEGs) and 198 differentially expressed metabolites (DEMs) were identified between T4 and T22. DEGs were significantly enriched in KEGG pathways related to carbohydrate, lipid, amino acid, and nucleotide metabolism, immunity, and signaling, while DEMs were significantly enriched in pathways associated with lipid and amino acid metabolism and membrane transport. The results indicated that cold stress altered RSCs’ metabolism and their innate immune system. This study provides valuable information to increase our understanding of cold stress responses in RSCs.

## 1. Introduction

Due to the significant economic value of red swamp crayfish (RSC), *Procambarus clarkii*, its global yield has sharply increased from 9.9 thousand tons in 2000 to nearly 2.5 million tons in 2020 [1]. In China, RSC farming is primarily concentrated in the provinces along the middle and lower reaches of the Yangtze River [2], as the optimal temperature for its growth and development ranges between 21 and 30 °C [3]. Low water temperatures, especially during the overwintering period, are known to be one of the challenges hindering the development of the crayfish aquaculture industry in northern China, particularly in northeastern China.

Water temperature is a crucial environmental factor for aquatic animals. Cold stress can affect various aspects of crustaceans and other aquatic ectotherms, including their behavior, growth and development, reproduction, metabolism, and immunity [4,5]. Crustaceans exposed to low temperatures may exhibit decreased activity and a reduction or cessation of feeding behavior. They may become immobile and can only survive for a few days [6,7,8]. Furthermore, low temperatures reduce the speed and quality of embryonic and gonad development, as well as overall growth [9,10]. Additionally, cold stress induces redox imbalance [11,12], lipid peroxidation, DNA damage, and alterations in osmolality [13]. At the molecular level, the expression of genes involved in the unfolded protein response (UPR) pathway, immunity, and apoptosis, is significantly changed under cold stress [5,14]. 

Omics technologies (e.g., transcriptomics, metabolomics, and proteomics) provide innovative approaches for exploring biological systems by screening and analyzing large datasets to characterize an organism under a specific condition [15,16]. To date, these technologies have been employed in several studies investigating the impact of cold stress on different crustaceans, including red claw crayfish (*Cherax quadricarinatu*), giant freshwater prawn (*Macrobrachium rosenbergii*), and Pacific white shrimp (*Litopenaeus vannamei*) [8,17,18]. Low-temperature stress could significantly downregulate the expression of numerous immune-related genes in the hepatopancreas and stimulate serine/threonine protein kinase activity [8], immunity, redox, and lipid metabolism pathways [17]. However, knowledge regarding the mechanisms underlying responses of RSCs to cold stress, particularly at the omics level, remains limited and requires further investigation. The hepatopancreas of crustaceans is a vital organ for coping with adverse stresses, as it plays a significant role in digestion, nutrient absorption and storage, immune defense, and physiological regulation [19]. Therefore, the hepatopancreas is one of the most important tissues for studying stress responses in crustacean species [8,20].

The primary objective of this study was to identify the key genes and metabolites involved in the response to cold stress and to elucidate molecular mechanisms underlying this response in RSCs by investigating the transcriptome and metabolome of the hepatopancreas in RSCs under cold stress treatment. This study could expand the current knowledge of crustacean responses to cold stress and provide valuable insights for enhancing its cold tolerance in aquaculture practices.

## 2. Results

### 2.1. Transcriptome Responses Under Cold Stress

The sequencing statistics for the six libraries are shown in Table 1. The number of raw reads ranged from 45.88 Mb to 57.69 Mb. The clean reads ranged from 45.54 Mb to 57.27 Mb. The values of Q20 and Q30 were above 98.57% and 95.61%, respectively, and the GC content exceeded 43.35% for all samples.

**Figure 1:**
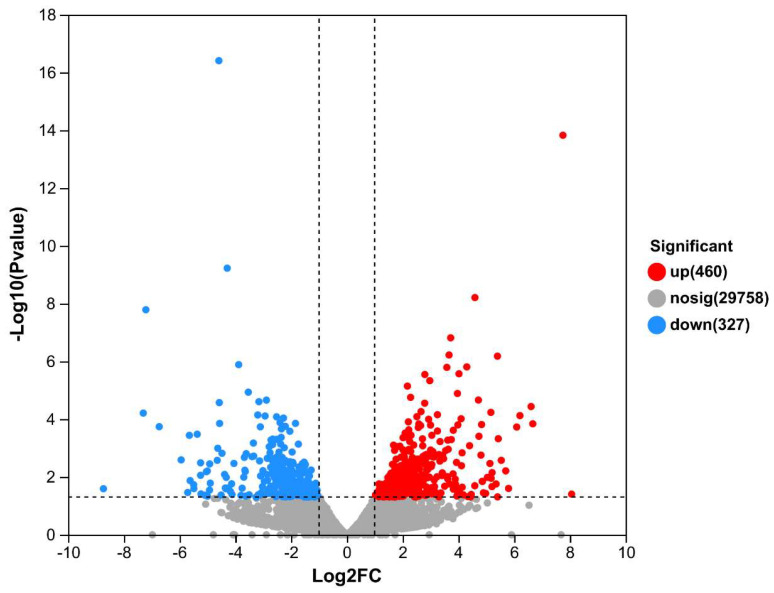
Volcano plot of DEGs between T4 and T22. Red dots represent significantly upregulated DEGs, blue dots represent significantly downregulated DEGs, and gray dots represent non-significant genes.

The number of differentially expressed genes (DEGs) between T4 and T22 was 787 (460 up and 327 down), as shown in Figure 1 and Supplementary Table S1.

**Figure 2:**
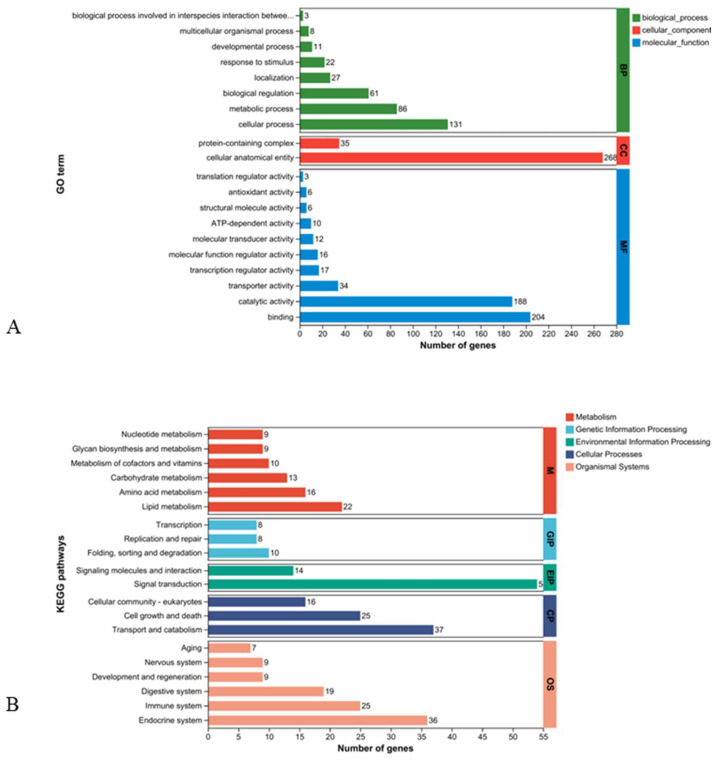
Bar plot of GO (**A**) and KEGG (**B**) enrichment analysis of DEGs.

GO and KEGG enrichment analyses were performed on DEGs. As for GO enrichment, most DEGs were enriched in a cellular anatomical entity (268) in cellular component, binding (204), and catalytic activity (188) in molecular function, as well as cellular process (131) and metabolic process (86) in biological process (Figure 2A). For KEGG enrichment, most DEGs were enriched in signal transduction (54) in environment information processing, transport, and catabolism (37) and cell growth and death (25) in cellular process, endocrine system (36), and immune system (25) in organismal systems, as well as lipid metabolism (22) in metabolism (Figure 2B).

**Figure 3:**
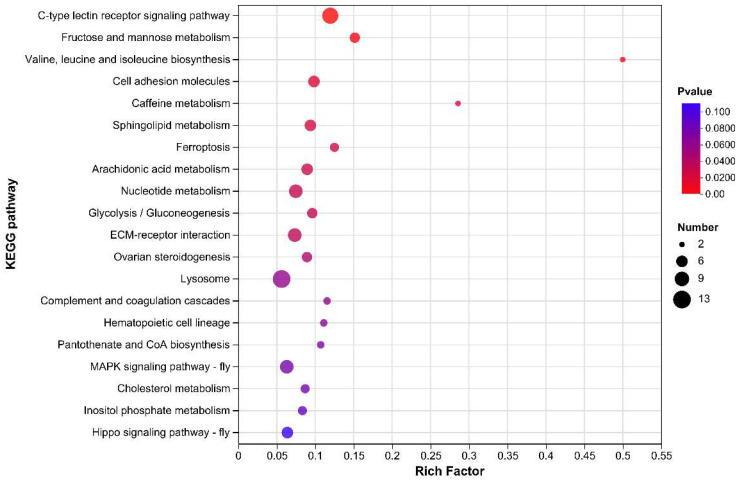
Bubble plot of KEGG pathway enrichment analysis of DEGs. The scatter size indicates the DEG number.

Pathway enrichment analysis showed that DEGs were significantly enriched in 12 pathways (*p* < 0.05), as shown in Figure 3. They included (1) C-type lectin receptor signaling pathway, (2) fructose and mannose metabolism, (3) valine, leucine, and isoleucine biosynthesis, (4) cell adhesion molecules, (5) caffeine metabolism, (6) sphingolipid metabolism, (7) ferroptosis, (8) arachidonic acid metabolism, (9) nucleotide metabolism, (10) glycolysis/gluconeogenesis, (11) ECM-receptor interaction, and (12) ovarian steroidogenesis.

**Figure 4:**
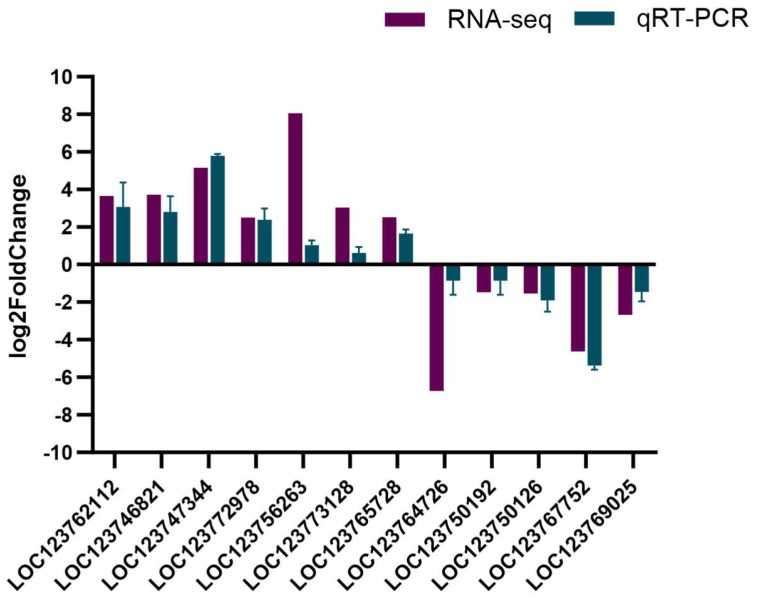
Validation of transcriptome sequencing data by qPCR.

To validate the reliability of the sequencing analysis, quantitative PCR (qPCR) was conducted on 12 DEGs using the *18S rRNA* gene as a reference gene. A consistent expression pattern was observed by comparing the qPCR results and the RNA-Seq data, confirming the reliability of RNA-Seq data (Figure 4).

### 2.2. Metabolome Responses Under Cold Stress

**Figure 5:**
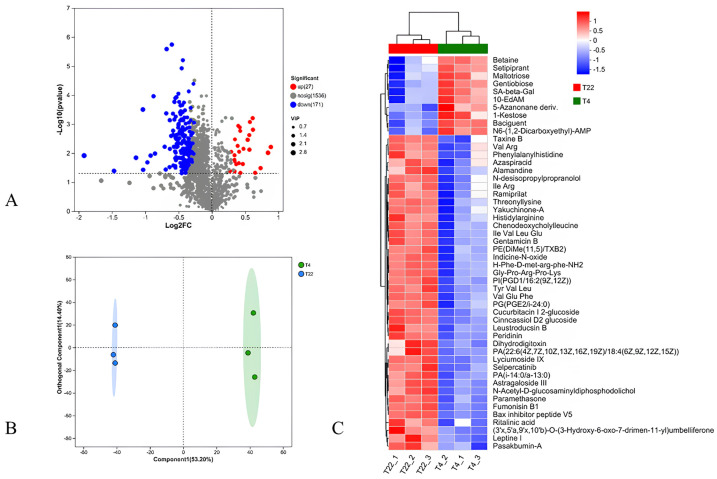
Differentially expressed metabolites between T4 and T22. (**A**) Volcano plot of DEMs between T4 and T22. (**B**) Score plots of OPLS-DA using DEMs. (**C**) Heatmap of the top 50 DEMs based on abundance.

Metabolome analysis was performed to investigate the metabolic alteration in the hepatopancreas of RSC under cold stress. Based on the criteria of VIP > 1.5 and *p* < 0.05, 198 metabolites were identified as differentially expressed metabolites (DEMs) between T4 and T22 (Supplementary Table S2), including 27 that were increased and 171 that were decreased (Figure 5A). The OPLS-DA analysis based on DEMs demonstrated a strong separation between T4 and T22 (R^2^ = 0.999, Q^2^ = 0.916), as shown in Figure 5B. Figure 5C shows the heatmap of the top 50 DEMs based on their abundance, where greater similarity within groups was observed between groups. 

**Figure 6:**
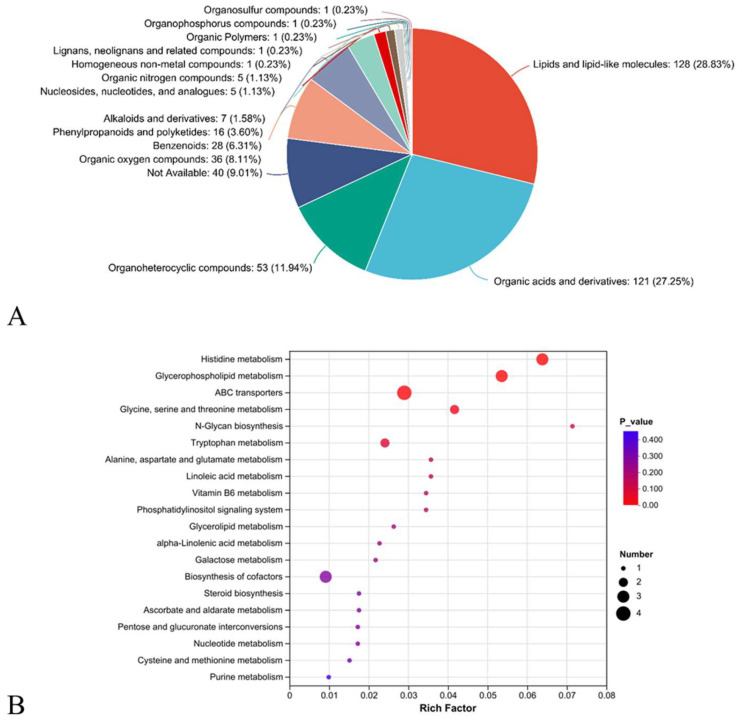
HMDB classification (**A**) and KEGG enrichment (**B**) of DEMs.

HMDB classification revealed that the DEMs were mainly classed into lipids and lipid-like molecules (51, 30.18%), organic acids and derivatives (47, 27.81%), and organoheterocyclic compounds (22, 13.02%; Figure 6A).

KEGG pathway annotation revealed that DEMs were enriched in four pathways (*p* < 0.05), including (1) histidine metabolism, (2) glycerophospholipid metabolism, (3) ABC transporters, and (4) glycine, serine, and threonine metabolism (Figure 6B). 

### 2.3. Correlation Between Transcriptome and Metabolome

**Figure 7:**
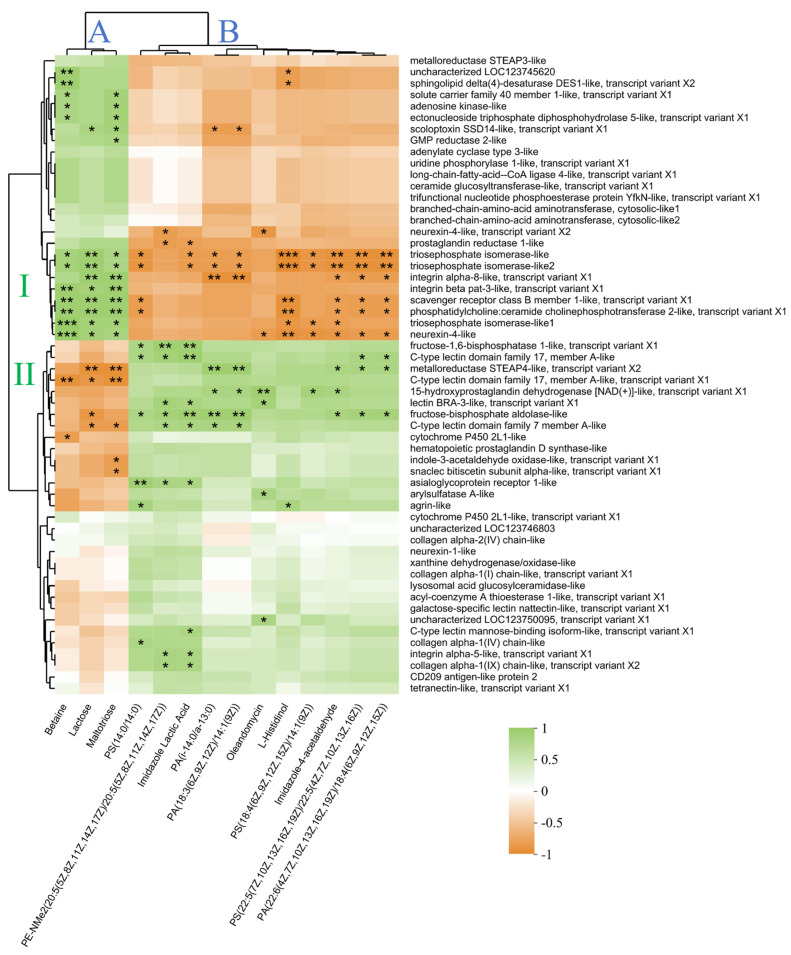
Spearman’s correlation heatmap between 56 SDEGs and 14 SDEMs. The color scale on the right shows the color partitioning of the different R values. * *p* < 0.05; ** *p* < 0.01; *** *p* < 0.001.

The DEGs and DEMs in the significantly enriched KEGG pathways were considered significant differentially expressed genes (SDEGs) and significant differential metabolites (SDEMs), respectively, resulting in a total of 56 SDEGs and 14 SDEMs. The key SDEGs and SDEMs are shown in Table 2. The correlation analysis between SDEGs and SDEMs is shown in Figure 7. The SDEMs were divided into Clusters A and B. Cluster A included betaine, lactose, and maltotriose enriched in the pathway ABC transporter. Cluster B included the seven phospholipid SDEMs and four other SDEMs mainly enriched in pathways including histidine metabolism, glycerophospholipid metabolism, and glycine, serine, and threonine metabolism. The SDEGs significantly correlated with the above SDEMs were mainly concentrated in two clusters (I and II, Figure 7). Cluster I mainly included SDEGs enriched in KEGG pathways of fructose and mannose metabolism and glycolysis/gluconeogenesis (three triosephosphate isomerase-like genes) and pathways of cell adhesion molecules and ECM-receptor interaction (integrin beta pat-3-like, integrin alpha-8-like, and neurexin-4-like). Cluster II mainly included DEGs enriched in the C-type lectin receptor signaling pathway (three C-type lectin genes and lectin *BRA-3*-like). Briefly, SDEGs in Cluster I mainly positively correlated with SDEMs in Cluster A but negatively correlated with Cluster B, while SDEGs in Cluster II exhibited the opposite pattern. 

## 3. Discussion

Water temperature is a crucial abiotic factor for crustaceans [4]. Therefore, understanding the molecular regulatory mechanisms of cold stress response in RSCs and analyzing their adaptative capacity is essential. Although RSCs are considered a warm-water species, they have a certain degree of tolerance to low temperatures [21,22,23]. In this study, RSCs were exposed to cold stress at 4 °C for 96 h, and transcriptomic and metabolomic analyses were conducted using hepatopancreas tissues to evaluate the effects of cold stress. A total of 787 DEGs and 198 DEMs were identified between the cold stress group (T4) and the control group without cold stimulation (T22). These DEGs and DEMs were significantly enriched in pathways related to metabolism, immunity, and other functions.

### 3.1. Cold Stress-Induced Alterations to Pathways Related to Metabolism

Low temperatures can directly affect the metabolism of crustaceans [4]. For example, cold stress altered genes and metabolites associated with carbohydrates, lipids, amino acids, and nucleotide metabolism in the Pacific white shrimp [18]. In this study, DEGs and DEMs between T4 and T22 were also significantly enriched in pathways related to carbohydrate metabolism. In the pathways of glycolysis/gluconeogenesis and fructose and mannose metabolism, fructose-1,6-bisphosphatase (FBP), and fructose-1,6-bisphosphate aldose (FBA) play key roles in gluconeogenesis [24,25]. In this study, their expressions were significantly downregulated in T4, indicating that the gluconeogenesis process was hindered in RSCs under cold stress. This result is consistent with similar studies on Pacific white shrimp [18,26]. Since gluconeogenesis is an energy-consuming process, ectothermic organisms are likely to reduce non-essential energy expenditure and prioritize survival at low temperatures [18].

Cold stress had a significant impact on lipid metabolism in this study. Sphingolipid metabolism was changed by cold stress in RSCs, which is consistent with the study in red claw crayfish [8]. Herein, the expression of sphingolipid 4-desaturase (*DES1*) was significantly upregulated in T4 compared to T22. Studies on red claw crayfish under low-temperature conditions have also identified the overexpression of *DES1* and Δ6 desaturase [27,28]. Similarly, increased desaturase activity has been reported in milkfish (*Chanos chanos*) and Nile tilapia (*Oreochromis niloticus*) exposed to low temperatures [29,30]. The overexpression of these desaturases would facilitate the conversion of saturated fatty acids (SFAs) to unsaturated fatty acids (UFAs). Since increased UFAs help maintain cell membrane fluidity at low temperatures [27], this overexpression supports the normal biological functions of RSCs under cold stress. DEGs were also significantly enriched in the arachidonic acid metabolism pathway (enrichment score = 2.587), which has been reported in kuruma shrimp (*Marsupenaeus Japonicus*) and Pacific white shrimp under cold stress [20,26]. In this pathway, T4 was characterized by the downregulation of cytochrome P450 family 2 genes (*CYP2*), which was consistent with the study of Pacific white shrimp [26]. In addition to metabolizing arachidonic acid to epoxyeicosatrienoic acids [31], *CYP2* plays a critical role in defending against environmental stress and pathogen infection in aquatic animals [32]. In studies on mud crabs (*Scylla paramamosain*), *CYP2* expression was significantly altered by *Vibrio parahaemolyticus* infection and ammonia stress, and interference with *CYP2* led to significantly higher malondialdehyde (MDA) content and increased mortality rate [32]. DEMs from metabolomic analysis were also enriched in the glycerophospholipid metabolism pathway, which is consistent with the study of giant freshwater prawns [33]. Glycerophospholipids are important biological fuels for energy supply [34]. Because crayfish tend to exhibit a fasting response when exposed to low temperatures [27], we discontinued feeding the RSCs in both T4 and T22 groups during the experiment. The reduction in these glycerophospholipids in T4 indicated that RSCs consumed them to increase energy production and maintain physiological homeostasis under cold stress.

Cold stress also altered amino acid metabolism in RSCs. In the DEMs, many tripeptides, mainly including branched-chain amino acids (BCAAs: valine, leucine, and isoleucine) were significantly decreased in the T4 group (Figure 5C). A similar decrease in leucine and isoleucine was also detected in the metabolites of kuruma shrimp under cold stress [20]. Meanwhile, overexpression of branched-chain aminotransferase (*BCAT*) in valine, leucine, and isoleucine biosynthesis pathways was detected [26,34]. *BCAT2*, which plays an essential role in the biosynthesis and degradation of branched-chain amino acids [35], has been reported to be upregulated in the hepatopancreas of Pacific white shrimp under cold stress [26]. We speculate that cold stress induced the decrease in the branched-chain amino acids, and stimulated the overexpression of *BCAT2*.

DEMs between T4 and T22 were also significantly enriched in KEGG pathways of histidine metabolism and glycine, serine, and threonine metabolism. According to the previous literature, metabolites in the histidine metabolism pathway were also altered in mrigal carp (*Cirrhinus mrigala*) and European seabass (*Dicentrarchus labrax*) [36,37]. It has been speculated that histidine metabolism may play critical roles in regulating adversity development, such as compensatory growth [37]. 

In this study, DEGs between T4 and T22 were significantly enriched in the nucleotide metabolism pathway, which has been reported in many fish and crustaceans under cold stress [18,38]. Nucleotides are of great importance to organisms, as they are involved in many biological processes, including DNA and RNA synthesis, energy storage, and metabolism, and other metabolic regulators [38]. In this study, the expression of xanthine dehydrogenase/oxidase-like (*XDH*), which is involved in hypoxanthine and xanthine degradation, was reduced when RSCs were under cold stress. Other genes, including GMP reductase 2-like (*GMPR*), 5′-nucleotidase (*NT5E*), adenosine kinase (*ADK*), and ectonucleoside triphosphate diphosphohydrolase 5/6 (*ENTPD5_6*), involved in purine synthesis, degradation, and salvage were upregulated in T4 group. The results indicate that cold stress altered the nucleotide metabolism in RSCs.

### 3.2. Cold Stress-Induced Alterations to Immune System 

In addition to metabolism-related pathways, cold stress also impacted immune system pathways in RSCs. Some C-type lectin receptor genes, including *DC-SIGN*, macrophage C-type lectin (*MCL*), and macrophage inducible C-type lectin (*Mincle*), were significantly downregulated in T4 compared with T22. In the previous literature, the downregulation of the C-type lectin gene has also been found in Pacific white shrimp and Nile tilapia exposed to low temperatures [26,39]. C-type lectin plays an immunomodulatory role in the innate immunity of crustaceans, including cell adhesion, pathogen recognition, and bacterial clearance [30,31,32,33,34,35,36,37,38,39,40,41,42]. Zhang et al. suggested that the overexpression of a C-type lectin in RSCs could significantly enhance the resistance to the bacteria *Vibrio anguillarum* [43]. In this study, the downregulation of C-type lectin receptor genes and other immune-related genes, such as *CYP2* mentioned previously, indicates that cold stress has a negative impact on immune competence and overall resistance to pathogens. Therefore, aquaculture management strategies, such as water treatment, strict quarantine measures, and clean feed, should be applied to prevent disease-causing agents.

### 3.3. Cold Stress-Induced Alterations to Other Pathways 

In the ECM-receptor interaction pathway, RSCs in T4 were characterized by the downregulation of collagen genes (*COL2A*, *COL4A*, and *COL9A*). Additionally, collagen abundance in T4 was significantly lower compared to T22, as shown by the metabolomic analysis. The reduction in collagen may indicate tissue damage in the hepatopancreas caused by cold stress. Previous studies have suggested that cold stress can cause morphological damage to the hepatopancreas of fish and crustacean animals [8,20,36] and lead to cell apoptosis [44]. In this study, DEGs were also significantly enriched in the ferroptosis pathway, which has been reported in the Nile tilapia exposed to low temperatures [45]. Ferroptosis is primarily caused by unrestricted lipid peroxidation and subsequent membrane damage [46]. Lipid peroxidation caused by cold stress has been identified in Pacific white shrimp [18]. In this study, long-chain acyl-CoA synthetase (*ACSL4*) and ferroportin (*FPN*) were significantly upregulated in the T4 group. *ACSL4* plays a role in the esterification of CoA to polyunsaturated fatty acids, thereby altering cellular lipid composition and increasing cellular susceptibility to ferroptosis [47]. High *ACSL4* expression is considered a marker of ferroptosis [48,49]. In this study, its overexpression might be related to the *FPN*, a plasma membrane iron export protein that plays a role in regulating intracellular iron concentration [50]. Previous studies in humans have shown that downregulated *FPN* induces ferroptosis by increasing iron concentrations and reactive oxygen species (ROS) levels [51], while upregulated *FPN* inhibits oxidative stress-induced ferroptosis [52]. In this study, higher expression of *FPN* suggested that RSCs may possess some capacity to inhibit the ferroptosis induced by cold stress. 

In conclusion, the transcriptomic and metabolomic of RSC hepatopancreas suggested that cold stress not only significantly affects the metabolism of carbohydrates, lipids, amino acids, and nucleotides in RSCs but also influences their innate immune system and induces ferroptosis. These impacts could significantly affect the functions of the hepatopancreas. The compromised immunity and the induction of ferroptosis may further exacerbate the vulnerability of RSCs under cold stress. Based on these findings, the following measures may enhance the cold tolerance of RSCs. Increasing dietary polyunsaturated fatty acids (PUFAs) and BCAAs may improve the cold tolerance of RSCs. PUFAs not only provide energy but also help maintain cellular physiological functions in cold environments. Previous studies showed that increasing the PUFAs in the diet significantly improved the survival rate of Nile tilapia under low temperatures [53]. Given the overexpression of BACT2 and the reduction in BCAAs in the T4 group, adjusting dietary BCAA proportions may improve the cold tolerance of RSCs. Earlier research demonstrated that optimized leucine improved growth performance in swimming crabs (*Portunus trituberculatus*) [54] and Pacific white shrimp [55] and increased antioxidant levels in the hepatopancreas of the swimming crabs [54]. Further investigations are necessary to confirm the effects of PUFAs and BCAAs on the cold stress tolerance in RSCs. Additionally, considering the compromised immunity of RSCs in the T4 group, minimizing their exposure to pathogenic factors is crucial. This study expands our knowledge of the response of RSCs to cold stress, which will be valuable for future research on the cold tolerance of RSCs. 

## 4. Materials and Methods

### 4.1. Cold Stress Treatment and Sample Collection

The RSCs were purchased in October 2023 from Shenyang Yurun Agricultural Products Wholesale Market, sourced from a grower in Qianjiang, Hubei. Prior to the experiments, RSCs were temporarily cultured in the aquatic animal lab of Shenyang Agricultural University under continuous aeration at 22 °C for one week. The crayfish were fed a commercial diet twice daily at 9:00 and 18:00, and one-third of the water was replaced daily. Dead crayfish, feed residues, and feces were cleaned up in a timely manner.

The RSCs (mean weight 17.57 ± 4.97 g; mean length: 8.37 ± 1.27 cm) were then randomly divided into two groups, with 20 RSCs in each group. To prevent mortality caused by a sharp temperature drop, one group was transferred to an incubator, where the temperature was gradually decreased from 22 °C to 4 °C in 12 h. The crayfish were then maintained at 4 °C for 96 h (a preliminary experiment indicated a mortality rate of approximately 50% after 96 h; T4). The other group was raised at 22 °C for 96 h (T22). To eliminate the effects of food intake, feeding was stopped for both T4 and T22 because the cessation of feeding behavior was observed in T4.

Six live RSCs were randomly collected from each group. The shell surfaces were rinsed with sterile water and then wiped using 75% ethanol. The hepatopancreas were immediately collected in a sterile environment. Each hepatopancreas was placed in a 2 mL cryogenic vial, submerged in liquid nitrogen, and stored at −80 °C. In each group, three of the hepatopancreas samples were used for transcriptomic sequencing, while the rest were used for metabolomic analysis.

### 4.2. Transcriptome Analysis

#### 4.2.1. RNA Extraction, Library Preparation, and Sequencing

Total RNA was extracted from hepatopancreas samples using QIAzol Lysis Reagent (Qiagen, Germantown, MD, USA) following the manufacturer’s instructions. Then, RNA quality was determined by 5300 Bioanalyser (Agilent) and quantified using the NanoDrop ND-2000 (Thermo Fisher Scientific, Wilmington, DE, USA). RNA purification, reverse transcription, library construction, and sequencing were carried out at Shanghai Majorbio Bio-pharm Biotechnology Co., Ltd. (Shanghai, China) according to the manufacturer’s protocols. RNA Purification Kit (Shanghai Majorbio Bio-pharm Biotechnology Co., Ltd., Shanghai, China) was used for RNA purification following the manufacturer’s instructions. For RNA-seq transcriptome library preparation, 1 μg of total RNA was processed following Illumina^®^ Stranded mRNA Prep, Ligation (Illumina, San Diego, CA, USA). Messenger RNA was isolated through the polyA selection method with oligo (dT) beads and fragmented using a fragmentation buffer. Double-stranded cDNA synthesis was conducted using a SuperScript double-stranded cDNA synthesis kit (Invitrogen, Carlsbad, CA, USA) with random hexamer primers. The resulting cDNA underwent end-repair, phosphorylation, and adapter ligation according to library construction steps. Target fragments of 300 bp were selected on 2% Low-range Ultra Agarose, and the library was amplified by 15 cycles of PCR using Phusion DNA polymerase (NEB). After quantification using a Qubit 4, sequencing was performed on the NovaSeq X Plus platform (Illumina, San Diego, CA, USA) with the NovaSeq Reagent Kit (Illumina, San Diego, CA, USA). 

#### 4.2.2. Quality Control and Read Mapping

The raw paired-end reads were trimmed and quality controlled by fastp [56] with default settings. The resulting clean reads were then individually aligned to the reference genome [57] in orientation mode using HISAT2 [58]. For each sample, the mapped reads were assembled using a reference-based approach by StringTie [59].

#### 4.2.3. Differentially Expression Analysis and Functional Enrichment

To identify differential expression genes (DEGs) between T4 and T22, the expression levels of each transcript were determined using the transcripts per million (TPM) reads method. Gene abundances were quantified using RSEM [60]. Differential expression analysis was conducted with DESeq2 [61] and DEGseq [62]. Genes were considered significantly differentially expressed if they met the criteria of *p* < 0.05 and |log2FC| ≧ 1. Functional enrichment, including GO and KEGG analysis, was performed to identify significant enrichment of DEGs in GO terms and metabolic pathways using *p* < 0.05. GO enrichment and KEGG pathway analysis were performed using Goatools and Python scipy software, respectively.

#### 4.2.4. Verification of the RNA-seq Results

To assess the reliability of the RNA-seq results, 12 genes were randomly selected from DEGs and validated using quantitative PCR (qPCR). Primers were designed using Primer-BLAST (https://www.ncbi.nlm.nih.gov/tools/primer-blast/, accessed on 29 January 2025) [63], and synthesized by Sangon Biotech (Shanghai) Co., Ltd. (Shanghai, China) The 20 μL reaction mixture contained 10 ng of DNA template, 1 μL of 10 μM forward and reverse primers, and 10 μL of ChamQ Universal SYBR qPCR Master Mix (Vazyme Biotech Co., Ltd., Nanjing, China). QPCR was performed on the QuantStudio3 system (Thermo Fisher Scientific, Foster City, CA, USA). The qPCR began with a pre-incubation phase at 95 °C for 30 s. DNA amplification was performed over 40 cycles under the following conditions: denaturation at 95 °C for 10 s, annealing at 60° for 30 s, and the specificity of amplicons was confirmed through melt curve and agarose electrophoresis. The primers’ details are provided in Table 3. The relative expression levels were normalized by the reference gene [*18S rRNA* gene (18S)] [64] through the 2^−ΔΔCt^ method [65].

### 4.3. Metabolomic Analysis

#### 4.3.1. Metabolite Extraction

Hepatopancreas samples (50 mg) were placed in a 2 mL centrifuge tube with a 6 mm diameter grinding bead. To extract metabolites, 400 μL of extraction solution [methanol/water = 4:1 (*v*:*v*)] containing 0.02 mg/mL of internal standard (L-2-chlorophenylalanine) was added. Samples were ground for 6 min (−10 °C, 50 Hz) using the Wonbio-96c frozen tissue grinder (Shanghai Wanbo Biotechnology Co., Ltd., Shanghai, China), followed by low-temperature ultrasonic extraction for 30 min (5 °C, 40 kHz). After 30 min at −20 °C for 30 min, the samples were centrifuged (15 min at 4 °C, 13,000 g), and the supernatant was transferred to the injection vial for LC-MS analysis.

#### 4.3.2. Quality Control Sample

To ensure system conditioning and quality control, a pooled quality control (QC) sample was created by mixing equal volumes of all samples. The QC samples were processed and analyzed in the same way as the test samples. These QC samples represented the entire dataset and were injected at regular intervals to monitor the consistency and stability of the analysis.

#### 4.3.3. LC-MS Analysis 

The LC-MS analysis of the samples was performed using a Thermo UHPLC-Q Exactive HF-X system (Thermo Fisher Scientific, San Jose, CA, USA) at Majorbio Bio-pharm Technology Co. Ltd. (Shanghai, China). The mobile phases included solvent A, which was 0.1% formic acid in a water mixture (95:5, *v*/*v*), and solvent B, composed of 0.1% formic acid in acetonitrile/isopropanol (47.5:47.5:5, *v*/*v*). The flow rate was set at 0.40 mL/min, with a column temperature of 40 °C and an injection volume of 3 μL.

#### 4.3.4. Data Analyses

The raw data from the LC/MS analysis were preprocessed using Progenesis QI software (Waters Corporation, Milford, MA, USA). Metabolite identification was carried out by searching databases, including the HMDB (http://www.hmdb.ca/), Metlin (https://metlin.scripps.edu/), and Majorbio Database. The resulting data matrix was subsequently uploaded to the Majorbio cloud platform (https://cloud.majorbio.com) for further analysis. Data filtering was applied to retain only metabolites detected in at least 80% of any sample group. Missing values were replaced with the minimum value, and metabolic features were normalized by summation. Sample mass spectrum peak intensities were normalized using the sum normalization method. Variables showing a relative standard deviation (RSD) greater than 30% in the quality control (QC) samples were excluded. The final data matrix was created through log10 transformation. Orthogonal partial least squares discriminant analysis (OPLS-DA) was conducted using the R package "ropls" (Version 1.6.2). Significant differences in metabolites were determined based on the variable importance in projection (VIP) score from the OPLS-DA model and the *p* values from Student’s *t* test. Metabolites with VIP > 1.5 and *p* < 0.05 were considered significantly different metabolites (DEMs). These DEMs were classified using the HMDB database and mapped to biochemical pathways through enrichment and pathway analysis using the KEGG database (*p* < 0.05). The “scipy.stats” Python package (https://docs.scipy.org/doc/scipy/, accessed on 29 January 2025) was employed for pathway enrichment, using Fisher’s exact test to determine statistically significant results. All analyses were conducted via the Majorbio I-Sanger Cloud Platform [66]. 

### 4.4. Correlation Between Transcriptome and Metabolome

The Spearman correlation coefficients between SDEGs and SDEMs were calculated and visualized by the corrplot package in R (1.6.2).

## 5. Conclusions

In this study, we conducted a comprehensive evaluation of the transcriptome and metabolome of the hepatopancreas of RSCs exposed to cold stress at 4 °C (T4) and 22 °C (T22: control) for 96 h. The results indicated that cold stress caused significant changes in gene expression and metabolic levels in RSCs. A total of 787 DEGs and 198 DEMs were identified between T4 and T22. DEGs were significantly enriched in KEGG pathways related to carbohydrate, lipid, amino acid, and nucleotide metabolism, immunity, and signaling, while DEMs were significantly enriched in pathways associated with lipid and amino acid metabolism, and membrane transport. Additionally, cold stress negatively impacted the innate immune system and induced ferroptosis, which significantly affected hepatopancreas functions. These findings could provide a better understanding of the molecular mechanisms of RSCs’ responses to cold stress and could benefit its aquaculture practices.

## Figures and Tables

**Table 1:** Quality control of the RNA-seq data.

Sample	Raw Reads	Clean Reads	Clean Read (%)	Q20 (%)	Q30 (%)	GC (%)	Total Mapped (%)
T22-1	45880024	45542106	99.26	98.66	95.77	48.01	43019610 (94.46%)
T22-2	57686400	57274276	99.29	98.64	95.77	45.97	53716548 (93.79%)
T22-3	54019958	53610324	99.24	98.63	95.74	46.01	49694951 (92.7%)
T4-1	50656502	50267000	99.23	98.57	95.61	43.35	46344494 (92.2%)
T4-2	56267340	55850560	99.26	98.62	95.76	45.14	51419269 (92.07%)
T4-3	47082160	46718294	99.23	98.63	95.74	47.29	43361392 (92.81%)

**Table 2:** The key SDEGs and SDEMs of RSCs associated with cold stress in some important pathways.

Category	Pathway	Gene/Metabolite	Description	Up/Down
Immune system	C-type lectin receptor signaling pathway	LOC123748590 (*CLEC17A*)	C-type lectin domain family 17, member A-like	down
		LOC123774641 (*MINCLE*)	galactose-specific lectin nattectin-like	down
		LOC123755747 (*DC-SIGN*)	CD209 antigen-like protein 2	down
		LOC123755745 (*BRA-3*)	lectin *BRA-3*-like	down
		LOC123750066 (*ASGR1*)	asialoglycoprotein receptor 1-like	down
		LOC123750185 (*MBL*)	C-type lectin mannose-binding isoform-like	down
		LOC123750192 (*CLEC7A*)	C-type lectin domain family 7 member A-like	down
		LOC123751783 (*TN*)	tetranectin-like, transcript variant X1	down
Carbohydrate metabolism	Glycolysis/gluconeogenesis	LOC123773396 (*TPI*)	triosephosphate isomerase-like	up
		LOC123760072 (*FBP*)	fructose-1,6-bisphosphatase 1-like	down
		LOC123768117 (*FBA*)	fructose-bisphosphate aldolase-like	down
Lipid metabolism	Sphingolipid metabolism	LOC123771454 (*ARSA*)	arylsulfatase A-like	down
		LOC123762036 (*CEGT*)	ceramide glucosyltransferase-like	up
		LOC123773128 (*DES1*)	sphingolipid delta (4)-desaturase *DES1*-like	up
		LOC123759095 (*GBA*)	lysosomal acid glucosylceramidase-like	down
		LOC123745757 (*SGMS*)	phosphatidylcholine/ceramide cholinephosphotransferase 2-like	up
	Arachidonic acid metabolism	LOC123760576 (*15-PGDH*)	15-hydroxyprostaglandin dehydrogenase [NAD (+)]-like	down
		LOC123767752 (*CYP2*)	cytochrome P450 2L1-like	down
		LOC123746626 (*HPGDS*)	hematopoietic prostaglandin D synthase-like	down
		LOC123765799 (*PTGR1*)	prostaglandin reductase 1-like	up
	Glycerophospholipid metabolism	HMDB0112860	PS (22:5(7Z,10Z,13Z,16Z,19Z)/22:5(4Z,7Z,10Z,13Z,16Z))	down
		HMDB0114400	PE-NMe2(20:5(5Z,8Z,11Z,14Z,17Z)/20:5(5Z,8Z,11Z,14Z,17Z))	down
		HMDB0012330	PS (14:0/14:0)	down
		HMDB0115412	PA (22:6(4Z,7Z,10Z,13Z,16Z,19Z)/18:4(6Z,9Z,12Z,15Z))	down
		HMDB0115811	PA (i-14:0/a-13:0)	down
		HMDB0112486	PS (18:4(6Z,9Z,12Z,15Z)/14:1(9Z))	down
		HMDB0114974	PA (18:3(6Z,9Z,12Z)/14:1(9Z))	down
Amino acid metabolism	Valine, leucine, and isoleucine biosynthesis	LOC123765728 (*BCAT2*)	branched-chain amino acid aminotransferase	up
	Glycine, serine, and threonine metabolism	HMDB0112860	PS (22:5(7Z,10Z,13Z,16Z,19Z)/22:5(4Z,7Z,10Z,13Z,16Z))	down
		HMDB0000043	betaine	up
		HMDB0012330	PS (14:0/14:0)	down
		HMDB0112486	PS (18:4(6Z,9Z,12Z,15Z)/14:1(9Z))	down
	Histidine metabolism	HMDB0003905	imidazole-4-acetaldehyde	down
		HMDB0003431	L-Histidinol	down
		HMDB0253418	imidazole lactic acid	down
Nucleotide metabolism	Purine metabolism	LOC123745620 (*NT5E*)	5′-nucleotidase	up
		LOC123761741 (*ADK*)	adenosine kinase-like	up
		LOC123767745 (*ENTPD5_6*)	ectonucleoside triphosphate diphosphohydrolase 5-like	up
		LOC123769025 (*XDH*)	xanthine dehydrogenase/oxidase-like	down
		LOC123765068 (*GMPR*)	GMP reductase 2-like	up
	Pyrimidine metabolism	LOC123762660 (*UDP*)	uridine phosphorylase 1-like	up
Signaling molecules and interaction	Cell adhesion molecules	LOC123769373 (*NRXN1*)	neurexin-1-like	down
		LOC123756722 (*ITGB*)	integrin beta pat-3-like	up
		LOC123746350 (*ITGA5*)	integrin alpha-5-like	down
		LOC123761742 (*NRXN4*)	neurexin-4-like	up
		LOC123770063 (*ITGA8*)	integrin alpha-8-like	up
		LOC123766564 (*NRXN4*)	neurexin-4-like	up
	ECM-receptor interaction	LOC123756722 (*ITGB*)	integrin beta pat-3-like	up
		LOC123746350 (*ITGA5*)	integrin alpha-5-like	down
		LOC123764756 (*AGRN*)	agrin-like	down
		LOC123770063 (*ITGA8*)	integrin alpha-8-like	up
		LOC123763235 (*COL9A*)	collagen alpha-1(IX) chain-like	down
		LOC123772781 (*COL4A*)	collagen alpha-2(IV) chain-like	down
		LOC123772701 (*COL4A*)	collagen alpha-1(IV) chain-like	down
		LOC123760044 (*COL2A*)	collagen alpha-1(I) chain-like	down
Membrane transport	ABC transporters	HMDB0255932	oleandomycin	down
		HMDB0000043	betaine	up
		HMDB0041627	lactose	up
		HMDB0038852	maltotriose	up
Cellular processes	Ferroptosis	LOC123768078 (*STEAP4*)	metalloreductase *STEAP4*-like	down
		LOC123760415 (*STEAP3*)	metalloreductase *STEAP3*-like	up
		LOC123767749 (*ACSL4*)	long-chain fatty-acid--CoA ligase 4-like	up
		LOC123760010 (*SLC40A1*)	solute carrier family 40 member 1-like	up

**Table 3:** Primers of qPCR in RSCs.

Gene ID	Gene	Description	Primer Sequence (5′-3′)	Product Size (bp)
LOC123762112	*cptp*	ceramide-1-phosphate transfer protein-like	F: CAGGACGCTGCTACGACTAC	116
			R: GTCCGTACAGCTCTGACACC	
LOC123746821	*se1L2*	protein sel-1 homolog 2-like	F: CTACGGTGATGGTGCCAAGT	103
			R: CCTCGCCTTGGTGATGTTCT	
LOC123747344	*-*	uncharacterized, transcript variant X2	F: GCCCGCATTTGTCGTGTC	80
			R: GCAGAAGCCTACACTGGAAAA	
LOC123772978	*rfx1*	*RFX*-like DNA-binding protein *RFX1*, transcript variant X1	F: GTGTCATGCCCTTGTTCTCTG	147
			R: ATTTTGCATCCGTGTTCCTGG	
LOC123773128	*des* *1*	sphingolipid delta (4)-desaturase *DES1*-like	F: GACCAGCGGGTGAGAGAAAA	87
			R: CACCAATCCCAGTACCCACC	
LOC123765728	*bcat*	branched-chain amino acid transaminase	F: GTGAGGACGCTCGGGATTTT	87
			R: ATCATCACTCCCAACCCGTC	
LOC123756263	*hsc70*	heat shock 70 kDa protein cognate 4-like	F: CCACTTACTCGGATACCAGCC	89
			R: ATTGTGCAGGTTTGTCAGCC	
LOC123764726	*elp4*	elongator complex protein 4-like	F: GACTTGCCTGCCTCAACAGA	117
			R: AACAGAAGTTGTGGGCTGGG	
LOC123750192	*clec7a*	C-type lectin domain family 7 member A-like	F: CCCCAAAGATCACCGAGAGG	122
			R: GGAGCAGCGTGTTGGATAGA	
LOC123750126	*clec17a*	C-type lectin domain family 17, member A-like, transcript variant X2	F: TTCAGCATGGCTCGCTTGT	128
			R: TTCATGGGCGTGTCGTGG	
LOC123767752	*cyp2*	cytochrome P450 2L1-like	F: CTGTTCATTGGTGGGACGGA	77
			R: ACCTCAGGGTACTGAGCCAT	
LOC123769025	*xdh*	xanthine dehydrogenase/oxidase-like	F: GAGGGACTTGCTTTCCCTCA	78
			R: GGTGTGGGCAGGTATCTTGT	
AF436001.1	*18s*	18S ribosomal RNA	F: CTGTGATGCCCTTAGATGTT	259
			R: GCGAGGGGTAGAACATCCAA	

## Data Availability

Transcriptomic data in this study have been deposited in the Sequences Read Archive (SRA) (https://www.ncbi.nlm.nih.gov/sra, accessed on 29 January 2025), the accession number of our submission is PRJNA1172931. The data that support the study findings are available from the corresponding author upon request.

## Supplementary Materials

The following supporting information can be downloaded at: https://www.mdpi.com/article/10.3390/ijms26031221/s1.
